# The Epigenetic Architecture of Classical Hodgkin Lymphoma: Lineage Erasure, Immune Escape, and Therapeutic Reprogramming

**DOI:** 10.3390/medicina62091740

**Published:** 2026-09-09

**Authors:** Matei Seleusan, Diana Cenariu, Adrian-Bogdan Țigu, Diana Gulei, Mădălina Nistor, Ximena Maria Muresan, Mihnea Zdrenghea

**Affiliations:** 1 Department of Personalised Medicine and Rare Diseases, Medfuture Institute for Biomedical Research, Iuliu Hațieganu University of Medicine and Pharmacy, 400349 Cluj-Napoca, Romaniagulei.diana@umfcluj.ro (D.G.); madalina.nistor@medfuture.ro (M.N.); ximena.muresan@medfuture.ro (X.M.M.); 2 Department of Haematology, Iuliu Hațieganu University of Medicine and Pharmacy, 400012 Cluj-Napoca, Romania; mzdrenghea@umfcluj.ro; 3 Department of Haematology, Ion Chiricuță Oncology Institute, 400015 Cluj-Napoca, Romania

**Keywords:** classical Hodgkin lymphoma, Hodgkin and Reed–Sternberg cells, epigenetics, lineage erasure, DNA methylation, histone deacetylation, Polycomb repression, immune evasion, non-coding RNA, therapeutic reprogramming

## Abstract

Classical Hodgkin lymphoma (cHL) is distinguished by a rare population of Hodgkin and Reed–Sternberg (HRS) cells that has largely extinguished its germinal center (GC) B-cell identity while maintaining survival, immune escape, and dependence on a highly organized tumor microenvironment (TME). This narrative review examines how interacting epigenetic mechanisms construct and stabilize this malignant state and evaluates whether it can be therapeutically reprogrammed. The relevant mechanistic and clinical literature available through 2026 was synthesized, encompassing lineage factor disruption, DNA methylation, histone modifications, Polycomb repression, chromatin-remodeling lesions, non-coding RNAs, antigen presentation, immune checkpoint regulation, and epigenetic therapies in cHL. Lineage erasure appears to be an actively maintained program produced by promoter hypermethylation, histone deacetylation, Polycomb-associated repression, altered histone demethylase activity, transcription factor antagonism, and somatic lesions affecting chromatin regulators. These mechanisms coexist with structural and transcriptional alterations involving antigen presentation and programmed death ligand 1/programmed death ligand 2 (PD-L1/PD-L2), while non-coding RNAs and extracellular vesicles provide an additional regulatory layer connecting HRS cells with immune and stromal components of the TME. Epigenetic repression is heterogeneous in its reversibility: partially methylated or deacetylated loci may remain pharmacologically responsive, whereas densely methylated, Polycomb-associated, or genetically entrenched states are less likely to be restored through isolated interventions. Histone deacetylase (HDAC) and DNA methyltransferase (DNMT) inhibitors can nevertheless alter tumor- and immune-related programs, supporting combinations with PD-1 blockade. Complete restoration of a physiological B-cell epigenome is unlikely; however, the HRS state remains therapeutically modifiable. Epigenetic therapy may therefore be most effective when used to reduce state stability and increase immune visibility rather than to achieve complete lineage reconstitution.

## 1. The B Cell That Erases Itself

Unusual among B-cell lymphomas, classical Hodgkin lymphoma (cHL) is characterized by a paradoxically minuscule fraction of malignant cells (comprising approximately 0.5–2% of the whole tumor mass within the affected lymph nodes) that have undergone profound lineage erasure to depart from the B-cell program and thus acquire neoplastic markers—a puzzling array of cell surface molecules pertaining to several hematopoietic lineages—that are essential for their survival [1,2]. This perturbed co-expression and identity are actively maintained by a cohort of epigenetic mechanisms [1,2,3]. The above-mentioned departure from the characteristic B-cell phenotype is sustained by the juxtaposition of DNA methylation, Polycomb repression, histone-modifying enzymes, lineage factor antagonism, viral mimicry, and signaling-dependent transcription. They all act in concert to simultaneously solve three problems that the aberrant B cell has to overcome in order to attain malignant progression: escape from germinal center negative selection, the reshaping of its immune visibility, and, most importantly, the establishment of a state that is difficult to classify via normal lineage rules. Therefore, epigenetic dysregulation in cHL should be understood as a state-building system that is continuously reinforced to stabilize the neoplastic cell [1,2,3].

Mononucleated Hodgkin and bi- or multinucleated Reed–Sternberg cells (collectively termed HRS cells) exhibit the marked downregulation of numerous B-cell transcription factors (e.g., OCT2, PU.1, and BOB.1—most plausibly mediated by the hypermethylation of their respective gene promoter regions, as no evidence points to genomic imbalances or chromosomal rearrangements within these CpG-rich sites), concomitant with the robust silencing of B-cell-specific genes via epigenetic mechanisms (including the general silencing of the immunoglobulin (Ig) loci) [1,3]. Together, these findings support a model in which the loss or functional inhibition of key B-cell transcription factors contributes to the widespread silencing of B-cell-specific genes, although the precise sequence and relative contribution of the cooperating regulatory mechanisms remain incompletely defined [1,3]. The loss of B-cell identity can be partly explained by epigenetic repression occurring within two contexts—DNA methylation and histone modification. The former often predominates in neoplastic cells to inhibit the transcription of tumor suppressor genes via either asynchronous monoallelic stochastic silencing or coordinated, homogeneous methylation that induces systematic biallelic silencing. The latter epigenetic mechanism, albeit acting in crosstalk with DNA methylation, inhibits target genes through chromatin compaction, transcription factor exclusion, and the recruitment of repressor complexes [3,4,5]. Interestingly, the silencing of several components of the B-cell receptor (BCR) signaling pathway might initially appear contradictory to the role of the BCR in sustaining B-cell survival. However, in cHL, as multiple anti-apoptotic cascades are simultaneously activated to prevent the cell from crossing the death-inducing threshold, silencing these particular genes might actually represent a selective advantage, especially since the BCR signaling hub is also involved in apoptosis regulation, with its absence—counteracted by several malignant pathways that will be discussed further—being beneficial to germinal center (GC) B cells en route to neoplastic progression [3]. Amplifications of certain chromosomal segments (most prominently the 9p24.1 amplicon) include genes encoding histone demethylase enzymes, complicating the apparent picture in which DNA hypermethylation is the main driver of oncogenic sustenance. As an example of this interplay between DNA and histone methylation, JMJD2C (encoded within the aforementioned amplicon) removes the repressive trimethylation of histone H3 lysine 9 (H3K9me3), which normally acts as a major epigenetic “off switch” that tightly packs DNA into silenced heterochromatin [6]. By removing these repressive H3K9me3 marks, DNA remains in a locally permissive chromatin state, enabling oncogenic drivers (such as *MYC*) to become available for transcription. Hence, the epigenome of the HRS cell state should be viewed through a nuanced, heterogeneous lens that takes into account the downstream loss of repressive histone methylation with ultimately pro-tumorigenic effects—as seen, for example, with the epigenetically mediated derepression of a long terminal repeat that contributes to the aberrant expression of the myeloid CSF1R receptor in HRS cells, which in turn sustains the subsequent expression of non-B-cell genes [2,6].

## 2. From Germinal Center Identity to Epigenetic Amnesia

### 2.1. Germinal Center Identity as a Controlled State

The intricate reactions occurring under physiological circumstances in GCs follow a predefined and well-preserved sequential order, being organized around the enhancement of activated B cells that have recognized their cognate antigen to maximize their binding capacity, culminating in the establishment of either plasma cells or long-lived memory B cells [7]. When somatic mutations yield nonfunctional Ig molecules (and, implicitly, nonfunctional BCRs), the cell is redirected toward irreversible demise unless rescue from apoptosis is provided in the form of neoplastic salvage pathways [7]. GCs—the microanatomical structures formed within B-cell follicles in secondary lymphoid organs after quiescent naïve B cells have been exposed to their cognate antigen—are polarized into two distinct areas with separate functional roles, namely the dark zone and the light zone, with B cells undergoing cyclic re-entry as part of their journey toward an optimal humoral immune response. Although this dark zone/light zone framework remains useful, recent single-cell transcriptomic analyses indicate that GC B cells occupy a more continuous spectrum of transcriptional states, including intermediate populations that cannot be assigned strictly to either compartment. Within this framework, dark zone centroblasts proliferate intensely and undergo somatic hypermutation driven by activation-induced cytidine deaminase (AID) so as to widen their antibody-binding capacity by generating randomly mutated variable Ig chains, while also changing to different isotype classes (class-switch recombination). The resulting less proliferative cells (centrocytes) migrate into the light zone to undergo affinity maturation—a process whereby these newly formed B cells test their novel receptor repertoire competitively on antigens bound to follicular dendritic cells (FDCs)—while simultaneously being aided by their cognate follicular T helper (Tfh) cells and thus guided by an antigen-driven Darwinian selection process [7,8,9]. Due to the high rate of mutational processes characterizing GC reactions, GC sites represent the most common histological compartments in which the malignant transformation of B cells occurs [8,9]. Several transcriptional modulators are transiently induced throughout this process: *MYC* exhibits bimodal expression, being both activated at early GC initiation and re-expressed in the light zone in B cells destined for dark-zone re-entry [8,9]. BCL6 is a transcriptional repressor affecting an intertwined network of target-gene effectors that drive GC formation, modulation, and subsequent sustenance. EZH2 is a component of Polycomb repressive complex 2 (PRC2) that catalyzes the methylation of lysine 27 on histone H3 (H3K27), with its expression in mature B cells restricted to the GC stage and its epigenetic silencing role implicated in lymphomagenesis through the sustained proliferation and prevention of B-cell differentiation. E2A is expressed in the dark zone to promote cyclin D3-driven cell cycle progression—all being part of a complex regulatory and signaling-dependent transduction module that is perturbed in neoplastic scenarios [8,9].

### 2.2. The Crippled B Cell and the Problem of Survival

Decades after its first characterization, cHL still presents a puzzling and unresolved scenario when it comes to deciphering the precise molecular events underpinning the trajectory from a clonal lymphoid precursor within a physiologically healthy GC setting to the establishment of malignant HRS cells. The smaller mononucleated compartment cycles slowly to generate post-mitotic multinucleated Reed–Sternberg (RS) cells that exhibit senescence-like features and have therefore ceased proliferating, while being highly involved in recruiting the perplexing mixture of non-neoplastic cells that forms the lymphoid, myeloid, and stromal components of the tumor microenvironment (TME), which accounts for 98–99.5% of the total tumor mass [2]. The ultimate stage of the neoplastic process, represented by RS cell formation, has been demonstrated to depend on disruptions in the shelterin protein complexes anchored at telomeric sites, leading to significantly shorter telomeres, telomere vulnerability, disorganized fusions, and gradual erosion, which culminate in DNA uncapping and the generation of the so-called “zebra” chromosomes of cHL [10]. The initial tumorigenic stages, however, are more difficult to track. It is well documented that HRS cells exhibit the marked activation of three crucial signaling pathways: nuclear factor kappa B (NF-κB), Janus kinase/signal transducer and activator of transcription (JAK/STAT), and mitogen-activated protein kinase/extracellular signal-regulated kinase (MAPK/ERK). These pathways provide a potent anti-apoptotic buffer, enabling survival under conditions that would generally trigger apoptosis in a GC B cell (such as loss-of-function Ig gene mutations that prevent the expression of the BCR, as seen in 25% of cHL cases, with the corresponding B cells being termed “crippled” due to their proximity to the apoptotic threshold under physiological conditions) [1,2]. Clonal Epstein–Barr virus (EBV) infection plays a crucial role during lymphomagenesis (being present in approximately 40% of cHL cases), especially in crippled B-cell cases, whereby signaling pathways enabling the survival of apoptosis-prone B cells are supplied through a type II latency program that substitutes for the missing BCR hub [10,11,12]. By contrast, EBV-negative cases depend on critical somatic genetic alterations and microenvironmental interactions that reinforce the constitutive activation of both canonical and non-canonical NF-κB pathways, JAK/STAT, and MAPK/ERK, all acting in concert to prevent the apoptosis of the malignant precursor [10,13]. The latency II expression pattern of EBV enables HRS precursors to express two crucial viral proteins: latent membrane protein 1 (LMP1) and latent membrane protein 2A (LMP2A). The former mimics CD40 receptor signaling and thereby substitutes for productive interactions with CD40L molecules expressed by Tfh cells within GCs during positive selection, while the latter contains a cytoplasmic module resembling that of the BCR complex and mediates tonic survival in cases carrying nonsense mutations in Ig genes [2,11]. Apart from genetic lesions that affect the orchestration of several signaling pathways, circulating tumor DNA (ctDNA) analyses have yielded a broader picture of the mutational landscape, including hits involving *EZH2*, which encodes the catalytic component of PRC2. Strong EZH2 expression has also been documented in HRS cells, while dysregulated EZH2 activity more broadly contributes to the transcriptional repression of B-cell differentiation and cell cycle programs and may impair antigen presentation pathways [14,15,16].

### 2.3. Lineage Erasure Is an Active Program

Importantly, HRS cells should be viewed neither as dedifferentiated blank cells nor as normal plasma cell intermediates but as a selectively reprogrammed hybrid state: the profound loss of B-cell identity is caused by interacting promoter methylation, chromatin repression, and transcription factor antagonism, producing a multilayered lock. Experimental evidence, discussed further below, shows that demethylating or acetylating agents cannot fully restore the B-cell phenotype despite, in principle, reversing components of the epigenetically induced state [5,17,18]. As hinted above, lineage loss is not produced by methylation alone: cHL tumor cells also lose their B-cell phenotype as a result of the aberrant expression of transcriptional regulators—normal differentiation toward the B-lymphoid lineage is controlled by a network of transcription factors, among which E2A (encoded by *TCF3*) and PAX5 are of significant importance, as they coordinate several B-lineage-specific transcriptional events responsible for normal terminal-state acquisition and maintenance of the differentiated phenotype [17]. E2A (encoding both E12 and E47) is antagonized by members of the inhibitor of differentiation (Id) family, which contain a helix–loop–helix (HLH) dimerization motif and, upon interaction with other basic helix–loop–helix (bHLH) transcription factors, such as E2A, prevent binding to the specific E-boxes (CANNTG sequences) responsible for transcription initiation. In turn, activated B-cell factor 1 (ABF-1; another type of bHLH that is upregulated as a result of NF-κB and activator protein 1 [AP-1], both constitutively active in HRS cells) includes a repression domain that prevents the activity of E47, the second protein encoded by *TCF3*. Together, Id and ABF-1 enforce competing interactions that permit the transdifferentiation and reprogramming of already committed B cells in cHL scenarios, showcasing the remarkable plastic potential of hematopoietic lineages and a possible route toward malignant transformation and multilineage marker expression [17]. One study identified an important mechanism through which the functional inhibition of E2A could be mediated: overexpression of the ABF-1 transcription factor and Id2 protein was associated with the complete absence of E47 homodimers [17]. As these antagonizing proteins inhibit E proteins from binding to their corresponding E-boxes (and multiple B-cell-specific gene expression programs depend on the recognition of these sequences), this finding was also consistent with the lack of transcription of E2A-controlled B-cell genes, such as *RAG1*, *RAG2*, *CD79A*, and *IGH* (the latter two being responsible for the expression of surface immunoglobulin and the successful establishment of the BCR complex). Importantly, the transfection of HRS cells with ABF-1 small interfering RNAs (siRNAs) did not restore endogenous B-cell-specific gene expression, therefore suggesting that stable epigenetic modifications could prevent the reactivation of the repressed genes, while the functional inhibition of B-cell gene expression could then be followed by the transcription of B-lineage-inappropriate programs that are otherwise inhibited [17,18,19].

It has been shown that cells in cHL adopt an aborted plasma cell differentiation program, exhibiting incomplete maturation accompanied by the extensive downregulation of B-cell antigens and no activation of genes typical of plasma cells [4]. Genome-wide cHL acetylation patterns were mapped via chromatin immunoprecipitation (ChIP) hybridization onto promoter tiling arrays (ChIP-chip), leading to the identification of 141 genes hypoacetylated in cHL compared with their hyperacetylated counterparts in B-cell lines (whereby the acetylation of lysines 9 and 14 of histone H3 [H3K9/14] is associated with gene activation), 23 of which encoded components of the BCR. The 17 genes found to be acetylated in cHL cell lines included the transcription factor that is important for plasma cell differentiation, *IRF4*, indicating possibly positive modulation [4]. Alongside this, numerous B-cell-specific promoters (e.g., *CD19*, *MS4A1*, and *CD79B*) exhibited histone deacetylation concurrent with DNA hypermethylation, which helps to explain their extinction within the malignant HRS expression program (although acetylation combined with demethylation alone does not restore expression, pointing to a more intricate network of epigenetic modifiers and transcription factor antagonism that act in concert to induce these effects) [4,5,17]. Importantly, *RYBP* (encoding the RING1 and YY1 binding protein), which constitutes an interacting partner for RING1A (part of Polycomb repressive complex 1 [PRC1]) and is not detected in normal B cells, was shown to be overexpressed in primary cHL cases and acetylated in cHL cell lines and thus appears to indirectly contribute to epigenetic silencing via Polycomb-mediated repression [4,20,21]. To further stabilize a deacetylated and promoter DNA methylation pattern, the trimethylation of H3K27 could serve as a durable silencing mechanism that sustains the inhibition of gene expression, as seen in B-cell promoters corresponding to several genes (*CD19*, *CD79A*, and *PAX5*) that showed this specific lysine methylation pattern deposited by the Polycomb group [4]. Another example of promoter alteration is represented by the tumor suppressor transcription factor KLF4, whose gene *KLF4* was found to be hypermethylated in primary cases of cHL and thus silenced—a finding supported by the fact that the enforced reactivation of *KLF4* promoted apoptosis in a BAK1-dependent manner (driven by the overproduction of BAK1 so as to exceed the sequestration capacity of BCL-XL), thus showing how epigenetic layers can ultimately prevent cellular death from occurring in GC-derived B-cell lymphomas [22]. Low *KLF4* transcription may precede its promoter hypermethylation and may be driven by high expression of the repressor NOTCH1 and epigenetic silencing of the activator PU.1. Furthermore, KLF4 negatively regulates markers that are aberrantly expressed in HRS cells, such as CXCL10, CD86, and ABF-1; hence, its absence coincides with the expected aberrant cHL phenotype [22,23,24].

**Figure 1:**
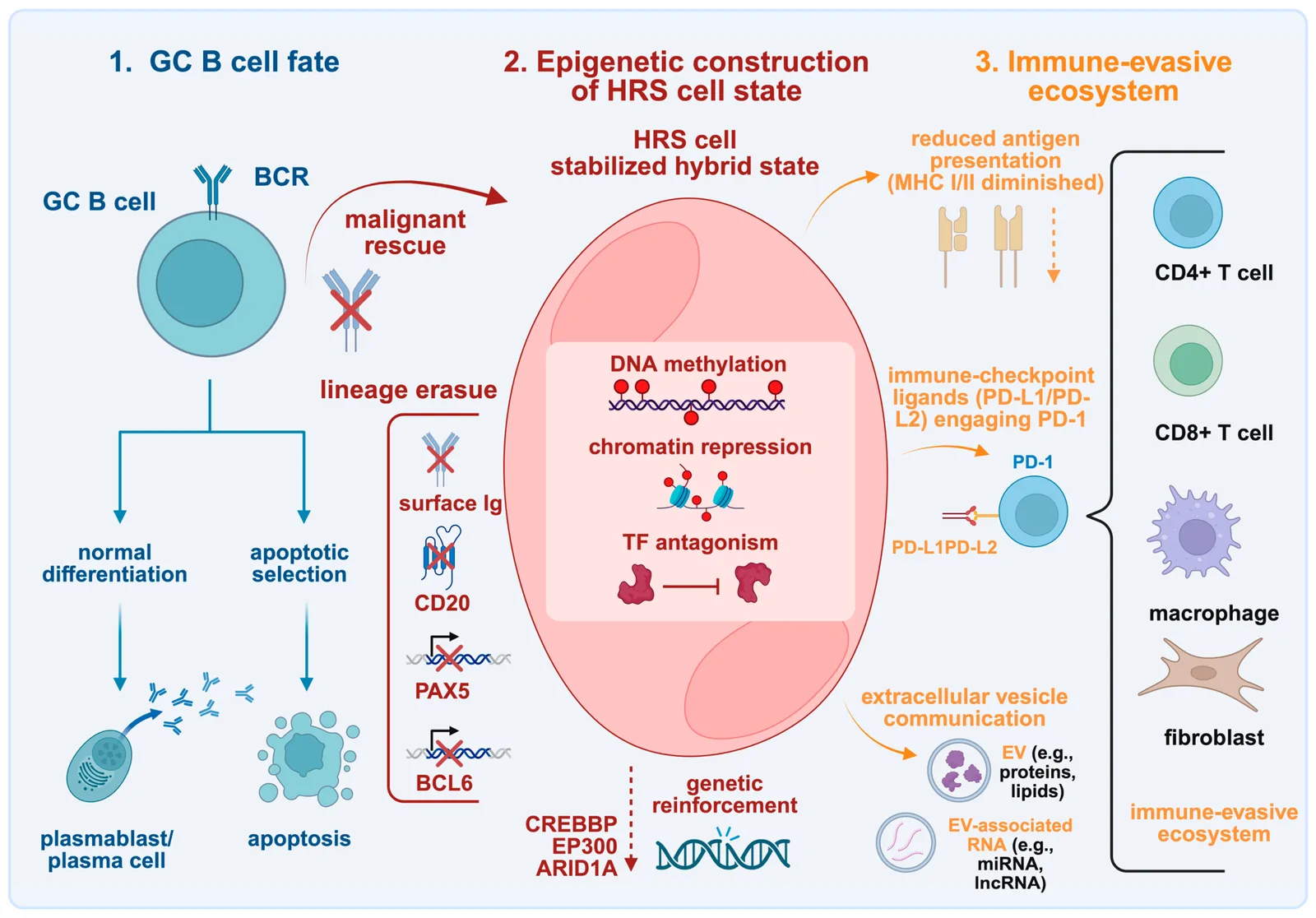
**Epigenetic construction of the Hodgkin and Reed–Sternberg cell state.** A germinal center B cell that has lost functional B-cell receptor signaling would normally be eliminated through the apoptotic pathway. In classical Hodgkin lymphoma, malignant rescue pathways permit survival, while an interacting network of DNA methylation, chromatin repression, and transcription factor antagonism extinguishes the physiological B-cell program—genetic lesions affecting chromatin regulators further reinforce the abnormal regulatory state. The resulting Hodgkin and Reed–Sternberg cell is not simply dedifferentiated but occupies a stabilized hybrid state characterized by lineage-inappropriate expression, reduced antigen presentation, immune checkpoint signaling, and active communication with immune and stromal components of the tumor microenvironment. Abbreviations: BCR, B-cell receptor; EV, extracellular vesicle; GC, germinal center; HRS, Hodgkin and Reed–Sternberg; Ig, immunoglobulin; lncRNA, long non-coding RNA; miRNA, microRNA; MHC, major histocompatibility complex; PD-1, programmed cell death protein 1; PD-L1, programmed death ligand 1; PD-L2, programmed death ligand 2; TF, transcription factor.

Another study identified the key B-cell transcription factor *ETS1* as frequently deleted and significantly downregulated in cHL cell lines and primary tumors: the research suggests that this genomic loss and subsequent silencing of *ETS1* disrupt the upstream regulation of the B-cell program, helping HRS cells to lose their B-cell identity and survive [25]. As previously mentioned, *IRF4* was found to be acetylated in HRS cells, exhibiting heightened activity. However, its function is structurally and functionally altered compared with normal B cells: a recurrent somatic mutation (p.Cys99Arg) in *IRF4* causes a functional shift that may contribute to the development of cHL. This mutation alters DNA binding, resulting in the loss of canonical binding alongside a neomorphic gain of binding to non-canonical composite elements (CEs) [26]. The altered IRF4-C99R protein blocks plasma cell differentiation and drives oncogenic gene expression through a novel AP-1-IRF composite element (AICE)-dependent mechanism (Figure 1) [26].

Importantly, the extent of B-cell lineage erasure is not uniform across all cHL cases. Lymphocyte-rich cHL may retain a comparatively stronger B-cell transcriptional program, with more frequent expression of factors such as OCT2, BOB.1, PAX5, and BCL6 than other cHL subtypes; this is consistent with less complete lineage silencing, although direct subtype-specific comparisons of DNA methylation and chromatin states remain limited [27]. Taken together, these findings indicate that lineage erasure in cHL is not a passive consequence of mere dedifferentiation but rather an actively maintained, multilayered program: transcription factor antagonism, loss of B-cell regulators, promoter hypermethylation, histone deacetylation, and Polycomb-associated repression act collectively to extinguish lineage identity while allowing aberrant survival programs to emerge.

## 3. The HRS Epigenome: Identity Written in Repression

### 3.1. DNA Methylation: Stabilizing the Silenced State

Methylation reflects one component of an interlocking repression system. Despite harboring functional and clonal Ig gene rearrangements, HRS cells globally lose the expression of their corresponding BCR [28]. This loss can be partly explained by a two-sided mechanistic story: premalignant B cells that have acquired nonsense mutations in their respective loci (within regulatory or coding sequences, occurring in 25% of cHL cases) are salvaged through neoplasia-related signaling pathways. In the remaining 75% of cases exhibiting no deleterious mutation, the inhibition of expression could be mediated mainly by the absence of critical transcription factors responsible for Ig heavy (H) and light (L) chain production, as well as by other epigenetic silencing mechanisms [28]. In both cHL cell lines and primary lymphoma cases, there is an established absence or substantial downregulation of critical factors involved in the control of Ig locus transcription (e.g., OCT2, BOB.1, and PU.1), and the ectopic expression of these factors does not restore endogenous Ig production, suggesting that other epigenetic players are involved in maintaining a repressed transcriptional state [29]. While a lack of crucial transcription factors is a robust explanation, one study examined whether transcription factors could bind to Ig-regulatory regions in both cHL cell lines and primary cultures and revealed a systematic inability of these factors to bind to the promoter, internal enhancer, and 3′ enhancer sites [28]. In addition, the study revealed H3K9 methylation in the Ig variable heavy-chain (IgVH) promoter region, strengthening the proposition that a methylated state can alter the chromatin structure and induce silencing at the level of Ig locus transcription [28]. Furthermore, the transcription factors enumerated above could be downregulated via the hypermethylation of their respective genes, in addition to deleterious mutations that disrupt their expression. A CpG island analysis of three representative genes (including one transcription factor, one glycoprotein component of the BCR, and one non-coding RNA) was performed to demonstrate this. *POU2AF1* promoters were found to be completely CpG-methylated, whereas the other two genes, *CD79A* and *TCL1A*, exhibited three different patterns of CpG DNA methylation in different cHL-related cell lines, showcasing a gradual repression-inducing event that is not binary (i.e., either methylated or demethylated); rather, it displays the gradual accumulation of methylation marks in a staged progression [30]. The first pattern was represented by CpGs that were densely methylated in flanking 5′ and 3′ DNA sequences, with CpGs in the central core promoter being only minimally methylated or completely unmethylated. The second pattern revealed an unmethylated core promoter flanked by methylated CpGs in the 5′ region, while CpGs located in the 3′ region were mostly unmethylated (significantly, this pattern could be reactivated by treatment with trichostatin A (TSA), a histone deacetylase (HDAC) inhibitor, indicating that histone deacetylation contributed to repression at these partially methylated loci) [30]. The third pattern consisted of intense CpG methylation along the entire promoter region, comparable with the methylation pattern representative of *POU2AF1* [30]. Thus, there appears to be a successive pathway of promoter methylation of varying functional importance and potential reversibility, which may help to explain the loss of typical B-cell gene expression alongside deleterious somatic mutations of transcription factors that lead to their absence during physiological B-cell maturation.

To further characterize the methylome of cHL, one study identified a total of 247 CpGs (corresponding to 209 genes) that were exclusively hypermethylated in HRS cells compared with control normal B-cell lines; these CpGs were mainly involved in the positive regulation of B-cell activation and regulation of T-cell differentiation, indicating an important function in the overall B-cell program (hypomethylating events were much rarer than in controls and were not examined further) [31]. These methylation patterns were strongly associated with de novo gene silencing, further suggesting that methylation-associated silencing is related to the extinguishment of the B-cell signature [31]. Additionally, since hypermethylation appears to be associated partly with the recruitment of DNA methyltransferases (DNMTs) to target genes by EZH2, a component of PRC2, the study also aimed to analyze whether the previously identified hypermethylated cHL-related genes were enriched for EZH2 or exhibited its predominant downstream mark, namely the trimethylation of H3K27 in centroblasts. This was indeed the case for 42% of the respective genes [31]. Therefore, to account for the remaining 58%, one must envisage additional epigenetic events that drive CpG hypermethylation apart from partial PRC2-mediated repression [31]. A useful comparative context is provided by mediastinal gray zone lymphoma (MGZL), which displays morphological and immunophenotypic features that are intermediate between nodular sclerosis cHL and primary mediastinal large B-cell lymphoma (PMBCL). DNA methylation profiling of microdissected tumor cells showed that MGZL likewise occupies an intermediate but distinct epigenetic position; notably, NS-cHL was characterized predominantly by de novo hypermethylation without detectable de novo hypomethylation, whereas both MGZL and PMBCL exhibited gains and losses of DNA methylation, indicating related but non-identical epigenetic programs [32].

The events preceding DNA methylation are of specific interest, as they could unravel the initial step in a multistage process that results in promoter silencing—an *NFATC1*-centered study provides a useful model for how promoter silencing may develop sequentially [33]. *NFATC1* is usually maintained in an active transcriptional state by antigen receptor signaling, and this is associated with histone H3 acetylation and H3K4 trimethylation, both of which act as activating chromatin marks that keep the promoter accessible. When receptor-dependent signaling was experimentally blocked in murine lymphoma cells, these activating marks were lost before any detectable CpG DNA methylation ensued. Established human cHL cell lines, however, showed both the loss of the active histone marks and extensive *NFATC1* promoter hypermethylation [33]. Therefore, the sequential model appears to rely on the withdrawal of receptor signaling, which first causes chromatin deactivation through histone deacetylation and loss of H3K4me3, after which promoter DNA methylation may occur to reinforce and stabilize long-term silencing [33]. Within CpG-rich islands, methylation occurs at the fifth carbon of the cytosine base, yielding 5-methylcytosine (5mC), which, as shown below, has considerable weight in influencing the transcriptional regulatory networks coordinating the B-cell program. Ten-eleven translocation (TET) family enzymes initiate active DNA demethylation by oxidizing 5mC to 5-hydroxymethylcytosine (5hmC) and subsequently to further oxidized derivatives, which can ultimately be replaced with unmodified cytosine through base excision repair; the levels of 5hmC have been shown to be depleted in several cancer types, serving as a potential epigenetic biomarker [34,35]. Its relevance to cHL appears particularly direct, as an immunohistochemical analysis demonstrated the near-universal depletion of 5hmC in neoplastic HRS cells across all 49 examined cases [36]. Moreover, the treatment of cHL-derived cell lines with vitamin C—a cofactor required for optimal TET enzyme activity—increased 5hmC levels while reducing cellular viability and inducing caspase activation, suggesting that impaired 5mC oxidation may contribute to the maintenance of the malignant HRS state and may remain at least partially reversible, although this evidence is currently limited to preclinical models [36].

### 3.2. Histone Modifiers and Polycomb: Building Repressive Memory

Polycomb group (PcG) proteins represent essential epigenetic repressors that maintain cellular identity, while also being implicated in lymphopoiesis, through two distinct multimeric protein complexes: PRC1 and PRC2. PRC2—composed of EZH1/2, EED, SUZ12, and RBBP4/7—initiates gene silencing via the deposition of the repressive histone mark H3K27me3 [37,38]. Canonical PRC1 complexes—composed of RING1A/B, PHC, CBX, and PCGF proteins such as BMI1—can then be recruited through the recognition of H3K27me3, whereas variant RYBP-containing PRC1 complexes can engage chromatin independently and deposit H2AK119ub1 upstream of, or in parallel with, PRC2 activity [38,39]. PRC1 catalyzes the ubiquitination of histone H2A at lysine 119 (H2AK119ub1), stabilizing chromatin compaction and blocking transcription; in malignant settings, the aberrant overexpression of EZH2 or other PRC2 subunits fuels neoplastic progression by silencing crucial tumor suppressor genes and enhancing proliferation and stem-like characteristics in various solid and hematological cancers [37,38,40,41]. In cHL, the upregulation of the core PRC2 catalytic subunit EZH2—along with other Polycomb components—is associated with a derailed epigenetic network that silences the B-cell-specific program, hence partly contributing to transformative events and abortive plasma cell differentiation [4]. It has long been known that HRS cells coexpress BMI1 and EZH2 proteins within their nuclei, which stands in contrast to normal follicular B cells, where their expression patterns are mutually exclusive (EZH2 is expressed primarily in rapidly dividing centroblasts, whereas BMI1 appears to be restricted to resting centrocytes). Therefore, coexpression is an intriguing epigenetic marker suggesting perturbed Polycomb-mediated repression during transformation and a deregulated cell cycle leading to aberrant PcG protein expression [42]. Furthermore, HRS cells and cHL-derived cell lines coexpress all core components of both PcG complexes (including the necessary binding partners of BMI1 and EZH2), a pattern that is consistent with the presence of both apparatuses and multiple downstream effects on gene expression modulation [43]. In transgenic mice, the upregulation of BMI1 induced the downregulation of p16^INK4A^ and p19^ARF^, increasing lymphoid proliferation and the subsequent development of lymphomas [44,45,46]. RYBP is the defining subunit of non-canonical, or variant, PRC1 that can be recruited independently of H3K27me3 and may deposit H2AK119ub1 upstream of, or in parallel with, PRC2 activity [39]. In a cohort of 321 cHL biopsy samples, RYBP was detected in 55% of cases, whereas it was absent in normal lymphoid subpopulations and lymphocyte-predominant Hodgkin lymphoma [21]. RYBP-positive cases exhibited an unfavorable treatment response and shorter overall survival [21]. The presence of BMI1 was also related to the increased expression of E2F6 in HRS cells (the respective protein representing another core subunit of variant PRC1); furthermore, PcG-protein expression exhibited a strong correlation with NF-κB activity, particularly in relation to MEL18, which belongs to the PCGF family and constitutes an active component of PRC1, being the closest direct homolog of BMI1, with MEL18 expression shown to depend on NF-κB activation [21,39,47].

HDACs are a family of highly conserved enzymes that remove acetyl groups from lysine residues on histones as well as non-histone proteins (including transcription factors, chaperone proteins, signal transduction mediators, and inflammatory mediators)—a process that generally induces heterochromatin formation and a repressed transcriptional state [48,49]. In cHL specifically, multiple HDAC isoforms are aberrantly overexpressed by HRS cells, disrupting the epigenetic balance and contributing to pathogenesis by altering both neoplastic HRS cells and several non-malignant reactive components of the surrounding inflammatory TME [50]. Because HDACs affect cell survival, growth, and proliferation, their dysregulation in malignant contexts renders them promising targets for cancer therapy, with hope that future agents will better modulate their specificity and selectivity so as to limit off-target toxicity. Class I enzymes were found to be highly expressed in HRS cells (consistent with the finding that patients with relapsed cHL had a 35% response rate upon administration of the class I HDAC inhibitor mocetinostat) and in cells within the TME, whereas the class II enzyme HDAC6 exhibited downregulation in lymphoid malignant settings and thus remains of questionable clinical relevance [51,52]. However, the promise of HDAC inhibitors remains, especially since some may show a dual effect by acting directly on neoplastic cells to inhibit proliferation while also exerting immunomodulatory effects on surrounding reactive non-malignant cells [51,52]. One study investigated the distribution of class I HDAC expression in cHL cases together with their associated tumor-infiltrating lymphocytes using a tissue microarray (TMA)—HDAC2 and HDAC3 were expressed in all analyzed HRS cell cases and in infiltrating lymphocytes within the reactive environment, while HDAC1 showed mean expression of 83% in the malignant population [53]. Another investigative approach identified the heightened expression of HDAC1, HDAC3, and the class IV enzyme HDAC11 in HRS cells, with lower expression of HDAC2 in the analyzed samples—importantly, higher HDAC1 expression correlated with shorter progression-free survival (PFS) and overall survival (OS) in the cHL patient cohort. Similarly, HDAC11 might be associated with decreased OS (the former had *p* < 0.05, while the latter exhibited greater uncertainty, with *p* = 0.05) [54].

While the previous sections have discussed the role of JMJD2C in establishing specific histone methylation patterns, KDM6B (JMJD3) is a histone demethylase enzyme that removes the trimethyl mark from H3K27 (being one of only two demethylases known to relieve these repressive marks by dissociating Polycomb-associated components) and appears to play a role in antigen-driven B-cell differentiation. KDM6B expression increases throughout the sequential stages that a B cell encounters en route to differentiation, helping to establish cellular identities related to the acquisition of memory and plasma cell features [55]. LMP1, part of the oncogenic program driven by EBV infection within precursor B cells undergoing transformation, has been shown to hijack and subvert transcriptional pathways responsible for physiological B-cell maturation via the modulation of KDM6B, which in turn induces aberrant modifications at the chromatin level. LMP1 appears to upregulate KDM6B expression in HRS cells, which was found to be overexpressed in 53% of primary cHL cases (of note, overexpression is identified in EBV-negative cases as well; hence, no definitive causal relationship can be drawn, as other regulatory mechanisms may contribute) [55]. As KDM6B naturally upregulates late-stage B-cell differentiation, its heightened expression within the cHL context reflects an interrupted transitional state, with HRS cells caught in an “epigenetic limbo”: late-stage differentiation regulators such as KDM6B are expressed, but core B-cell identity transcription factors have been lost, thereby producing phenotypic dedifferentiation [55]. Furthermore, KDM6B has been shown to induce additional transcriptional changes that do not depend on the removal of repressive trimethyl marks; hence, these layers of increased complexity require further examination [56]. Additionally, strong KDM4B/KDM4D (JMJD2B/JMJD2D) expression is associated with an aggressive cHL phenotype and is also linked to radioresistance [57]. A cHL-focused analysis of KDM4 expression suggests that strong cytoplasmic KDM4B and KDM4D staining identifies a subset of patients with limited-stage cHL and inferior relapse-free survival. KDM4D was seen most notably among those receiving involved-field radiotherapy who subsequently developed in-field relapse—raising the possibility that hypoxia-responsive KDM4B and DNA double-strand break repair-associated KDM4D contribute to treatment resistance and may serve as prognostic and potentially predictive biomarkers, although these findings require further independent validation (study cohort, *n* = 91; in limited-stage disease, 5-year relapse-free survival was 71% with strong versus 94% with lower KDM4D expression, *p* = 0.046; among limited-stage patients receiving involved-field radiotherapy, the corresponding rates were 60% versus 97%, *p* = 0.007) [57].

### 3.3. Genetic Lesions That Entrench an Epigenetic State

Genomic analyses of microdissected HRS cells have identified a series of critical mutations in cHL that also affect crucial epigenetic regulators: most studies predominantly focus on mutations affecting the NF-κB and JAK/STAT pathways, where somatic lesions have been extensively reviewed, while *B2M* alterations are among the most commonly identified inactivating modifications affecting β2-microglobulin expression and thus the cellular expression of major histocompatibility complex (MHC) class I molecules required for effective antigen recognition [2,58,59,60,61]. In refractory cases, mutations affecting *TP53* were of significant relevance, as their abundance is well known to be associated with chemoresistance in a variety of cancers [62]. The most frequently mutated genes identified in a large study, however, were *EP300* and *CREBBP*, both of which encode epigenetic regulators (the former representing a histone acetyltransferase involved in chromatin remodeling, while the latter is a master epigenetic regulator that also functions as a histone acetyltransferase, primarily mediating gene activation through open chromatin configuration) [62]. This finding is strengthened by additional evidence that histone acetyltransferases can act as tumor suppressors controlling MHC class II expression and immune evasion [63]. In this context, *CREBBP* and *EP300* alterations are best interpreted as putative loss-of-function or hypomorphic mutations rather than gain-of-function events within the broader framework of acetyltransferases as tumor suppressors: their impaired function would be expected to decrease histone acetylation and disrupt transcriptional programs, enabling derailed repression to occur.

Analyses of recurrent mutations, copy number alterations, and structural variants indicate that complementary immune evasion mechanisms coexist in cHL, with much of the mutational burden concentrated in EBV-negative cases, in which malignant survival and immune escape depend more strongly on disrupted genetic configurations that substitute for EBV-derived signaling [64]. Recurrent gains and amplifications of 9p24.1 correlate with the overexpression of programmed death ligand 1 and programmed death ligand 2 (PD-L1/PD-L2), thereby limiting effective immune clearance, while the same amplicon also reinforces the perturbation of the JAK/STAT pathway through increased dosages of *JAK2*, which can further augment PD-L1/PD-L2 transcription [65,66,67,68]. These mechanisms are complemented by mutations or deletions affecting *B2M* and human leukocyte antigen B (*HLA-B*), as well as structural alterations of class II major histocompatibility complex transactivator (*CIITA*), which disrupt MHC class I or class II antigen presentation and consequently reduce the immune visibility of HRS cells [64]. Importantly, the identification of predominantly truncating and splice-site mutations in *ARID1A*, a tumor-suppressive component of the switch/sucrose non-fermentable (SWI/SNF) chromatin remodeling complex, establishes an additional link between the genetic and epigenetic architectures of immune escape, as impaired chromatin remodeling may alter the accessibility of regulatory regions controlling antigen presentation, inflammatory signaling, and checkpoint expression [64]. Thus, genetic lesions in cHL do not act independently of the epigenome but help to establish the transcriptional and chromatin context through which immune-evasive programs become continuously expressed and stabilized.

Using targeted next-generation sequencing (NGS) panels, one study compared chemosensitive and chemorefractory tissue samples from patients with cHL and further reinforced the thesis that pathogenic variants of two critical epigenetic regulators (*CREBBP* and *EP300*) occur frequently in chemorefractory HRS cells and are retained within clonally expanding populations positively selected by first-line treatment (hence strengthening the idea that continuous aberrant epigenetic disruption contributes to disease progression and worsened prognosis) [69]. Specifically, *CREBBP* appeared to be the most frequently mutated gene in refractory or relapsed cHL samples, affecting 60% of patients in the analyzed cohort [69]. This alteration was accompanied by additional lesions in *ARID1A* and *EP300*, supporting a distorted epigenetic framework that disrupts essential transcriptional programs and may contribute to chemoresistance (the predominance of *CREBBP* alterations is not surprising, as *CREBBP* is centrally involved in modulating cellular fate throughout GC lymphomagenesis) [69,70].

### 3.4. What Is Reversible, and What Is Not?

To answer the central question posed by this section, it is necessary to delineate rapidly reversible repression from stable epigenetic memory: loss of histone acetylation and signaling-dependent activating marks may remain pharmacologically responsive, while extensively hypermethylated DNA and Polycomb-associated chromatin states are more likely to establish persistent repression that is refractory to targeted epigenetic therapies. As illustrated throughout the previous sections, reversibility is locus-specific and is generally unlikely to fully restore the regulatory state that preceded the malignant transformation of precursor GC B cells. Copy number alterations and somatic mutations that affect chromatin regulators impose an additional limitation, as these lesions cannot be reversed by the mere reopening of compacted chromatin (although some of their downstream transcriptional consequences could, in principle, remain pharmacologically modifiable).

The removal of an individual repressive mark should not be viewed as equivalent to restoration of the regulatory state that defined the precursor GC B cell before it underwent neoplastic transformation—chromatin accessibility should instead be assessed in the context of transcription factor availability, DNA methylation, histone modifications, and the overall nucleosomal architecture. Reopening a locus may be insufficient when the underlying transcriptional circuitry required for its reactivation has been extinguished—this is particularly relevant in cHL, where DNA demethylation, histone reacetylation, and the ectopic expression of individual factors have been shown to be inadequate for the full reconstruction of the physiological B-cell program, indicating that B-cell lineage repression is maintained through several reinforcing mechanisms of varied repressive magnitude [5,17,18,71,72]. There appears to be a hierarchy of therapeutic responsiveness across loci (although this remains inferential): promoters mainly regulated by histone deacetylation or partial CpG island methylation may retain a certain degree of plasticity, whereas densely methylated loci (particularly those embedded within Polycomb-associated chromatin) are likely to be more resistant to monotherapies. Resistance could be further reinforced by the loss of activating histone marks and the absence of essential transcription factors. This sequence is partly illustrated by the regulation of *NFATC1*: the withdrawal of receptor-dependent signaling first leads to the loss of activating histone acetylation marks and H3K4me3, after which DNA methylation may stabilize the resulting repressed state [30,31,33,37,38,40,41,71,72]. Reversing DNA methylation alone would likely be insufficient to restore the upstream signals that previously maintained transcription; similarly, increasing TET activity and 5hmC levels in HRS cells may demonstrate that part of the abnormal methylation landscape remains chemically modifiable, but it does not re-establish complete B-cell identity—genetic lesions affecting *CREBBP*, *EP300*, or *ARID1A* may create a durable (and potentially irreversible) barrier by sustaining aberrant chromatin remodeling states [33,36,62,63,64,69,70,71,72].

Epigenetic therapies may be most useful for sensitizing target cells, not for establishing full lineage restoration: DNMT and HDAC inhibitors can reactivate tumor antigens, enhance their presentation, and alter suppressive myeloid populations or exhausted cytotoxic T-cell states within the inflammatory milieu of cHL. These effects may render HRS cells more readily recognized by the immune system and more vulnerable to immune checkpoint blockade, although their magnitude is likely to remain context-dependent [71,72,73,74].

The HRS epigenome therefore comprises transient repression, stable chromatin memory, and genetically altered (and thus potentially permanent) regulatory states; importantly, the same layered architecture that extinguishes B-cell identity also shapes the antigens, immune checkpoints, and secreted signals through which HRS cells are recognized by the immune system, as discussed in the following section. The principal mechanisms stabilizing the HRS cell state and their relative potential for reversal are summarized in Table 1.

## 4. Immune Escape as a Potentially Epigenetically Maintained State

### 4.1. Antigen Presentation: Structural Loss and Selective Visibility

Antigen presentation failure in cHL is heterogeneous: while specific tumors carry irreversible structural defects, others may retain components that remain transcriptionally or epigenetically regulable. In cHL, HRS cells evade antitumor immunity by employing a variety of mechanisms: these include enhanced PD-L1 signaling (driven by copy number alterations of the 9p24.1 amplicon) and perturbed antigen presentation (sustained by lesions in *B2M*, leading to the loss of β2-microglobulin (β2M)/MHC class I protein complex expression in 79% of cHL cases), accompanied by the loss of MHC class II expression in 67% of cases, with the former being associated with shorter PFS [75]. Furthermore, inactivating alterations of the MHC class II transactivator *CIITA* were observed in cHL cases [76]. Notably, some cases retain β2M/MHC class I and MHC class II expression on the cell surface, albeit at substantially lower levels—these cases might be attributed to monoallelic loss or heterozygous inactivating mutations affecting the corresponding loci, or to upstream deficiencies in the transcriptional regulators maintaining their functions (e.g., NLRC5 and *CIITA*) [75,76]. *CIITA* in cHL is affected by both chromosomal in-frame fusion mechanisms and genomic breaks, which disrupt its physiological function; therefore, recurrent rearrangements of *CIITA* represent an intriguing genetic mechanism underlying tumor–microenvironment interactions, as these alterations consequently drive the downregulation of MHC class II expression and the overexpression of PD-L1/PD-L2, contributing to escape from immunosurveillance [76]. Additionally, a single-nucleotide polymorphism array analysis revealed destructive homozygous deletions within *CD58* in cHL cell lines, which led to the loss of expression of the corresponding cell surface glycoprotein, which binds the CD2 receptor and mediates interactions between malignant cells and CD8+ T cells, as well as natural killer (NK) cells [78].

While the direct chromatin-level mapping of MHC class II repression in HRS cells remains limited, the frequency of MHC class II loss exceeds that of identifiable *CIITA* rearrangements or MHC region deletions (structural lesions in *CIITA* alone cannot explain the full prevalence of MHC class II deficiency, as they occur in only 15% of cases), potentially suggesting that additional epigenetic mechanisms may contribute [76,79]. Relevant precedents are provided by mechanistic studies in two other GC-derived non-Hodgkin B-cell lymphoma settings, where loss of *CREBBP* depleted H3K27 acetylation at MHC class II regulatory regions, thereby allowing unopposed repression by BCL6–SMRT–HDAC3 complexes [80]. Furthermore, *CIITA*-negative lymphoma cells displayed reduced activating histone modifications at the *CIITA* promoters and the partial restoration of both *CIITA* transcription and MHC class II expression following HDAC inhibition, supporting the view that immune visibility can represent a chromatin-dependent phenotype rather than solely the consequence of irreversible genetic loss (this plausible epigenetic framework requires further direct validation in primary HRS cells) [77].

### 4.2. PD-L1/PD-L2 as Genomic, Transcriptional, and Bidirectional Signals

In the cHL setting, tumor cells exploit the programmed cell death protein 1 (PD-1) axis in various ways: apart from the amplicon leading to a copy number increase in immune checkpoint ligands, HRS cells exhibit constitutive AP-1 expression, its presence being correlated with an AP-1-responsive enhancer within *CD274* (the gene encoding PD-L1), where AP-1 binding enhances transcription [81]. Additionally, EBV status influences the expression pattern of PD-L1, as virally infected HRS cells induce ligand expression as a direct result of the oncogenic function of LMP1, which acts in concert with JAK/STAT-dependent promoter activity and the previously mentioned AP-1-associated enhancer. All mechanisms converge on the maintenance of the immunosuppressive state that characterizes the surrounding TME via the attenuation of T-cell receptor (TCR) signaling [81]. The tandem AP-1 binding sites are docking zones for both components of the AP-1 complex (c-JUN and JUNB), and enhancer activation led to augmented PD-L1 promoter-driven luciferase expression, indicating that the regulatory sites of the gene encoding PD-L1 are positively modulated within the HRS context. Surprisingly, there appears to be a mutually exclusive relationship between the presence of 9p24.1 amplification and EBV status, supporting the hypothesis that each mechanism represents an alternative method of PD-L1 induction [81]. Furthermore, there appears to be a reverse signaling mechanism that sustains the HRS state through inputs received via PD-L1 expressed in cHL cell lines. The engagement of this ligand with an agonistic monoclonal antibody increased cell survival and proliferation while also reducing apoptosis in the analyzed cells [82]. In addition, both membrane-bound and soluble forms of PD-1 (such as those found in the serum of patients with cHL) are capable of inducing this peculiar reverse signaling pathway, mediated by MAPK activation and ultimately leading to increased mitochondrial oxidation [82]. Since effective PD-1 blockade therapy also involves proper neoantigen presentation (and HRS cells downregulate the MHC complexes responsible for interaction with incoming effector cells), the activation of cytotoxic CD8+ T cells within the TME accounts for some of the consequences induced by this therapeutic modality (although it should be noted that CD4+ T cells acquire cytotoxic functions upon removal of the PD-1 brake, as they are intimately associated with malignant target cells). Hence, other mechanisms may contribute upon removal of the PD-L1/PD-L2–PD-1 interaction. One previously unexplored role of PD-L1 overexpression is that of providing a growth advantage to HRS cells by increasing the spare respiratory capacity (with malignant cells being highly dependent on mitochondrial metabolism) and downregulating apoptosis-related proteins [82,83]. An important role within the immune checkpoint ligand context pertains to tumor-associated macrophages (TAMs), found in close proximity to the sparse neoplastic population: beyond intrinsically expressing PD-L1 (which contributes to the immunosuppressive properties of the TME by inhibiting PD-1-expressing CD4+ T cells), macrophages display a phenomenon termed trogocytosis, whereby the ligands are transferred from HRS cells onto macrophages, enriching the population of PD-L1/PD-L2-expressing monocytes [84].

Although the amplification of 9p24.1 underpins the genetic basis upon which the overexpression of PD-L1/PD-L2 is supported, checkpoint abundance remains transcriptionally plastic [65,66,67,68]. In cHL cell lines, administration of the class I HDAC inhibitor mocetinostat increased both NF-κB and PD-L1 protein expression, indicating that chromatin-modifying therapy can further reshape the checkpoint phenotype superimposed upon the underlying copy number lesion [85]. Whether this effect reflects the direct remodeling of *CD274*-regulatory chromatin or indirect activation through parallel signaling pathways remains unresolved. The question of whether PD-L1 expression may also be transcriptionally regulated by as-yet-unidentified factors within upstream acetylation-sensitive networks deserves additional attention and mechanistic validation in primary HRS cells.

### 4.3. Non-Coding RNA as a Cross-Layer Regulator and Extracellular Signal

Beyond genomic and chromatin-level mechanisms of immune escape, evasion in cHL is also regulated post-transcriptionally through non-coding RNAs. By modulating transcript stability, translation, and intercellular signaling, microRNAs (miRNAs) and long non-coding RNAs (lncRNAs) can reinforce inflammatory signaling, potentially aiding the recruitment or polarization of immune cells within the TME [86,87].

It is well established that miRNAs can modulate hematopoietic lineage differentiation and affect a variety of cellular functions, with their expression patterns influencing pathophysiological scenarios such as chronic lymphocytic leukemia [88,89]. HRS cells exhibit high expression of *BIC* (a non-coding gene that encodes miR-155) in more than 90% of cases, with the possibility that T cells surrounding the sparse malignant population trigger enhanced *BIC* expression in HRS cells [90,91]. NF-κB appears to play a role in the induction of *BIC* expression, as the promoter region of the gene harbors a putative binding site for nuclear factors activated upon signaling through the NF-κB axis. Furthermore, miR-155 potentially alters the inducible T-cell co-stimulator (ICOS)–ICOS ligand (ICOSL) interaction by modulating ICOSL translation, a possibility that is strengthened by the presence of ICOS-positive T cells within the TME, while the loss of its respective ligand may influence the subsequent immune response [90,91,92]. As the transcription factor PU.1 represents another target of miR-155, it can be speculated that *BIC* expression contributes to the absence of this critical protein for early B-cell differentiation in cHL by downregulating its respective transcript [93,94]. miR-148a acts as a tumor-suppressive miRNA within the cHL setting, and a study utilizing primary HRS cell samples showed that epigenetic inactivation via CpG island methylation represents a plausible scenario whereby this particular non-coding RNA is downregulated (therefore, epigenetic silencing in cHL not only affects protein-coding genes but also contributes to the deregulation of miRNA expression). Its promoter was located within a known hypermethylated CpG-rich region, but the functional effects of its attenuated expression require further investigation [95]. It has been described that miR-148a acts within a regulatory circuit involving the NF-κB pathway, and the epigenetically mediated downregulation of this non-coding RNA could result in enhanced pathway activation, which is essential for lymphomagenesis in cHL [96]. Furthermore, miR-148a contributes to normal B-cell maturation, being expressed during physiological B-cell activation and promoting plasma cell differentiation; hence, the absence of miR-148a may represent one mechanism through which derailed differentiation occurs in cHL [97]. When compared with normal GC B cells, cHL cell lines exhibited the significant differential expression of 84 miRNAs, among which miR-24-3p was consistently overexpressed (a finding that was further validated in primary HRS cells)—the functional inhibition of miR-24-3p reduced cell growth and led to an increase in apoptotic remnants (at least partly through the derepression of the cell cycle inhibitor *CDKN1B*), suggesting that this particular miRNA contributes to the sustenance of the malignant HRS cell state [98].

Apart from miRNAs (which mainly affect target transcript and mRNA stability in a repressive manner), lncRNAs also play a role in cHL, exhibiting greater functional variety by affecting both the transcription and translation of their targets and acting as versatile molecular switches, with the exact activating or repressive effect depending on their structure, location, and, most importantly, the molecular partners to which they bind [99]. One study identified a distinct lncRNA expression program in cHL, with 475 lncRNA loci differentially expressed between HRS cells and normal GC B cells—among these, three showed HRS cell-specific expression in primary tissue samples, supporting their potential roles as tumor-specific biomarkers [100]. Furthermore, the identification of 107 spatially associated lncRNA–mRNA pairs (together with the preferential nuclear localization of selected candidates) suggests that some of these transcripts could contribute to the malignant state through cis-regulatory effects on neighboring genes [100].

Circulating extracellular vesicle (EV)-associated miRNAs (including miR-24-3p, miR-127-3p, miR-21-5p, and miR-155-5p) are enriched in active cHL, decline upon treatment, remain elevated in refractory disease, and rise again during relapse, thus supporting their use as biomarkers of the active tumor burden. Although a causal effect on immune cell polarization remains speculative, elevated EV-miR-122-5p at diagnosis was associated with increased circulating CD4+ T cells in pediatric cHL and was further related to relapse and inferior survival [101,102,103]. Independently of their RNA cargo, HRS-derived EVs have been shown to alter the secretory phenotypes of cancer-associated fibroblasts (CAFs), with CD30-enriched vesicles stimulating interleukin-8 (IL-8) release from eosinophils and granulocytes, demonstrating EV-mediated communication without proving that these effects are RNA-dependent [104,105]. Evidence more specifically implicating extracellular RNAs is provided by EBV-associated lymphoma models, in which exosomal BART miRNAs induced an immunoregulatory macrophage phenotype. Collectively, these studies suggest that extracellular non-coding RNAs may connect the HRS cell state with the immune and stromal components of the TME, although direct RNA cargo-mediated immune reprogramming by primary HRS cells remains to be functionally validated [106].

## 5. Therapeutic Reprogramming

The question of whether epigenetic therapies merely injure HRS cells, prime immune responses, or genuinely alter the state established by neoplastic chromatin abnormalities is of great interest, as it could inform future synergistic combinations that offset treatment-related toxicities and improve the design of more precise therapies targeting specific components of the malfunctioning regulatory pathways underpinning malignant cell maintenance.

### 5.1. HDAC Inhibition: Chromatin Perturbation Without Full Lineage Restoration

In the context of HDAC inhibitors, it is clear that acetylation-dependent programs are therapeutically vulnerable; however, the responses registered so far fail to show the establishment or restoration of pristine B-cell identity. A phase II study examined 129 patients with relapsed or refractory cHL after autologous stem cell transplantation and found that the administration of panobinostat (a potent pan-HDAC inhibitor targeting classes I, II, and IV and thus acting with limited selectivity) produced tumor reductions in 74% of cases, with an estimated 1-year OS rate of 78% [107]. Importantly, panobinostat treatment did not show cross-resistance to prior chemotherapeutic regimens, indicating its continued efficacy even in heavily pretreated patients [107]. However, the study did not investigate whether these positive effects were related to direct action on the HRS compartment or to the immunomodulation of the surrounding TME in favor of antitumor immunity. Another phase II study in patients with relapsed or refractory cHL utilized mocetinostat (an oral isotype-selective HDAC inhibitor targeting classes I and IV) and revealed a decrease in tumor burden in 81% of cases in which at least two cycles of therapy had been administered (most plausibly, the mechanism through which mocetinostat acts—as demonstrated in cHL-derived cell lines—is through the upregulation of p21, downregulation of STAT6, and activation of a caspase-dependent apoptotic pathway) [108,109,110]. Furthermore, mocetinostat could alter the efficiency of the antitumor immune response by decreasing the expression and secretion of thymus and activation-regulated chemokine (TARC), as well as by upregulating the OX40 ligand on the surfaces of HRS cells [109,111]. The former is actively secreted by HRS cells to construct a protective cellular shield by recruiting immune suppressors such as regulatory T cells (Tregs) and T helper 2 (Th2) cells into the TME, while the latter is usually suppressed in tumor settings, as its presence drives T-cell proliferation and enhances cytotoxicity. Importantly, mocetinostat treatment reduced serum TARC levels in most patients, with a positive effect on the clinical response, hence strengthening the claim that TARC may represent a useful in vivo biomarker and should not be viewed as being confined solely to effects seen in cHL-derived cell lines [108,109,111]. An additional phase II study analyzed the responses of patients with relapsed or refractory cHL to entinostat (an isoform-selective inhibitor primarily targeting class I HDAC enzymes), whereby a tumor reduction was observed in only 58% of cases [112]. Earlier in vitro studies suggested that entinostat exerts potent antiproliferative and immunomodulatory effects through p21 upregulation, the suppression of anti-apoptotic proteins, and the modulation of TARC expression; these properties may also contribute to the proposed synergy between HDAC inhibition and PD-1 blockade by reshaping myeloid-derived suppressor cell activity, increasing PD-L1 expression, and potentially reconfiguring the abnormal inflammatory TME [113,114,115,116].

Altogether, HDAC inhibition reveals that immune checkpoint blockade therapy and antibody–drug conjugates targeting CD30 might be augmented by chromatin-modifying modalities; however, the rationale for designing the most effective synergistic strategy likely depends on patient-specific oncogenic lesions and an in-depth analysis of case-specific epigenomic abnormalities, heightening the need for better patient-centered combinatorial treatments.

### 5.2. DNMT Inhibition and PD-1 Blockade: Immune Priming as Reprogramming

DNMTs are enzymes that establish and maintain cytosine methylation at CpG dinucleotides, with potent effects on transcriptional repression at many promoter-associated loci; their pharmacological inhibition can promote DNA hypomethylation, thereby reactivating silenced genes (such as those related to antitumor immunity) [117]. Combining DNMT inhibition with PD-1 blockade therapy (which prevents the engagement of PD-1 with its cognate immune checkpoint ligands expressed by the malignant population) represents a rational strategy in cHL to increase HRS cell immunogenicity and simultaneously restore immune effector activity.

Decitabine is a cytidine analog that traps and depletes DNMT1, promoting passive DNA demethylation. A phase II study enrolled patients with relapsed or refractory cHL who had previously undergone conventional treatment and administered a combination of decitabine and camrelizumab (anti-PD-1 treatment) to primarily assess complete remission (CR): the CR rate reached a remarkable 71% in the combination setting, much higher than with camrelizumab alone [118]. Therefore, methylation inhibition appears to enhance PD-1 blockade-mediated T-cell rejuvenation and partially restore antitumor immunosurveillance [118]. While PD-1 blockade can reverse the exhausted phenotype of T cells actively interacting with inhibitory immune checkpoint ligands expressed by both the malignant population and particular components of the inflammatory milieu, exhausted T cells possess a distinct epigenomic profile compared with effector and memory T cells (which could lessen the potency of PD-1 blockade over time) [119]. Therefore, the addition of decitabine could represent a pathway through which both tumor cells and lymphocytes become remodeled, although the particular regulatory networks that are reactivated remain unresolved. Another similar phase II study confirmed the same observation, yielding a CR rate of 79%, with combination treatment exhibiting the greatest potency in subgroups with relatively larger tumor burdens and those pretreated with three or more prior lines of therapy (importantly, the CR induced by combination therapy was directly associated with an increase in the percentage of peripheral central memory T cells) [120]. One study went a step further, concentrating on the select number of cases in which combination therapy was ineffective due to primary resistance, as well as those in which relapse or progression still occurred despite the synergistic modality. The proposed triplet regimen comprised chidamide (an HDAC inhibitor targeting class I and IIb enzymes), decitabine, and the anti-PD-1 antibody camrelizumab, yielding a CR rate of 50% [121]. Nonetheless, all enrolled patients exhibited therapeutic responses, with the triplet regimen shown to activate a diverse array of tumor-reactive CD8+ T cells while also inhibiting STAT1/3 signaling, which in turn led to the suppressed proliferation of interleukin-21-positive (IL-21+) CD4+ T cells (known to participate in the formation of inhibitory rosette structures around the HRS population and to upregulate anti-apoptotic genes through a positive feedback mechanism) [121]. The principal phase II studies evaluating epigenetic and epi-immunotherapeutic strategies in relapsed/refractory cHL are summarized in Table 2.

**Figure 2:**
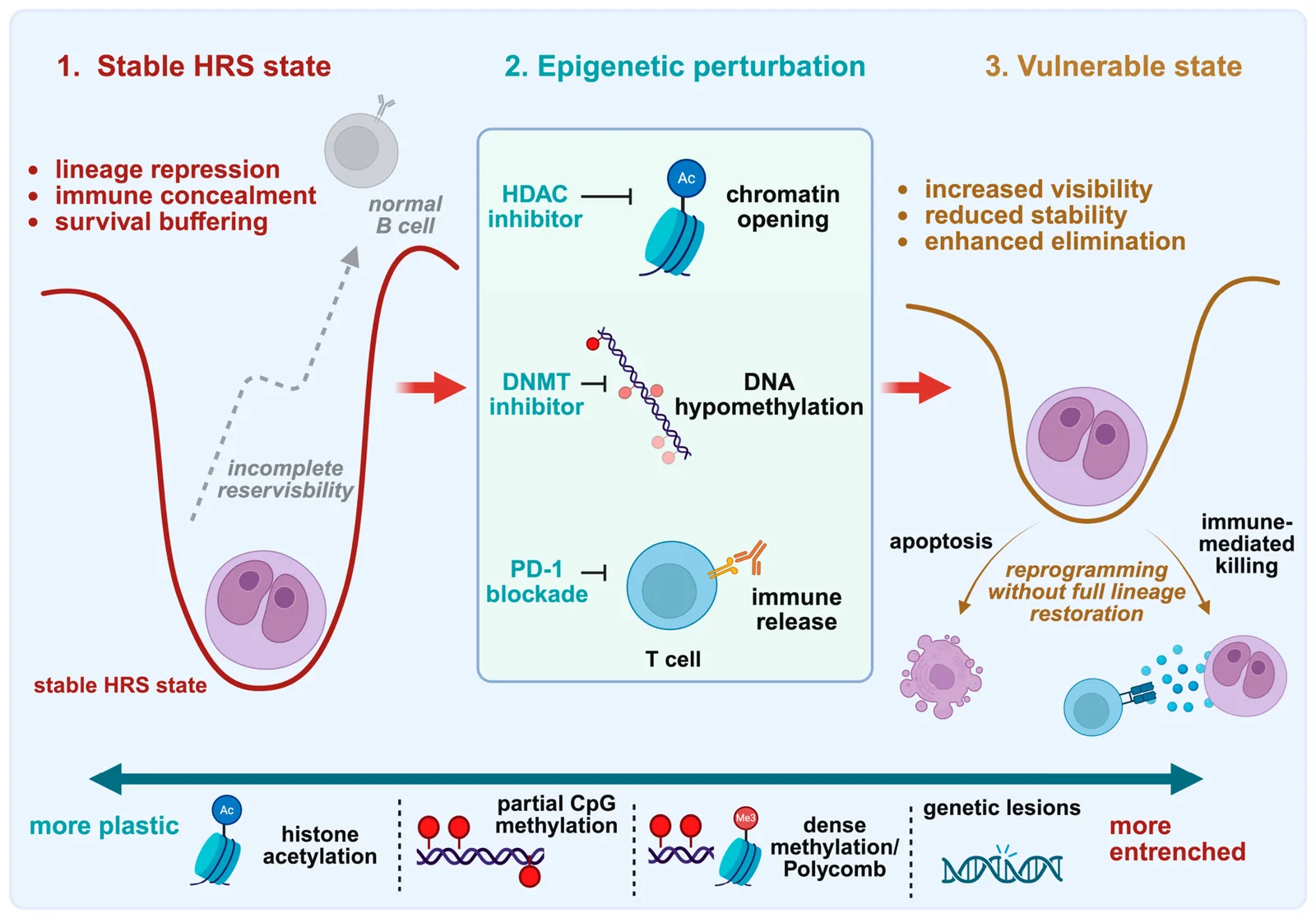
**Therapeutic displacement of the HRS epigenetic state.** The Hodgkin and Reed–Sternberg cell phenotype is stabilized by interacting transcriptional, chromatin, and genetic mechanisms that create a deeply entrenched malignant state—histone deacetylase and DNA methyltransferase inhibition can perturb selected regulatory programs, while programmed cell death protein 1 blockade releases immune inhibition at the tumor–immune interface. These interventions are unlikely to reconstruct a pristine B-cell epigenome, particularly where essential transcription factors have been extinguished or genetic lesions continuously reinforce abnormal chromatin regulation. Their principal therapeutic value may instead lie in reducing state stability, modifying immune checkpoint and antigen-related programs, and increasing susceptibility to apoptosis or immune-mediated elimination. The degree of reversibility is likely to differ across loci, with deacetylated or partially methylated regions remaining more pharmacologically plastic than densely methylated, Polycomb-associated, or genetically disrupted states. Abbreviations: CpG, cytosine–phosphate–guanine; DNMT, DNA methyltransferase; HDAC, histone deacetylase; HRS, Hodgkin and Reed–Sternberg; PD-1, programmed cell death protein 1.

All these findings reinforce the idea that novel optimizations in epigenetic–immunotherapy regimens could ultimately lead to longer-lasting responses in more patients with disease that has relapsed or is refractory to conventional treatment schemes, but the rationale for drug design should be better informed by the chromatin reconfigurations that accompany the supplementation of immunotherapy with epigenetic agents (Figure 2).

## 6. Conclusions

The epigenetic architecture of cHL should be viewed as an interlocking system through which a GC-derived B cell extinguishes its physiological identity, survives despite the loss of functional BCR signaling (essential for movement away from the apoptotic threshold during GC reactions), and acquires an immune-evasive phenotype. This state is not mere dedifferentiation. Instead, it is an identity built by the convergence of several abnormalities, including transcriptional repression, chromatin remodeling, and genetically fixed regulatory alterations, that act in concert to stabilize the hybrid HRS cell phenotype. Importantly, the same architecture underpinning the suppression of B-cell identity also contributes to reshaping the tumor’s immune visibility (and subsequent bidirectional communication with the surrounding TME), indicating that lineage erasure and immune escape are interconnected consequences of the same malignant program.

Therapeutic rewriting attempts are therefore unlikely to reconstruct a pristine B-cell epigenome, especially when essential transcription factors have been perturbed or when lesions involving *CREBBP*, *EP300*, and *ARID1A* continuously reinforce aberrant chromatin states. Nevertheless, the potent activity of HDAC and DNMT inhibitors (especially in combination with immune checkpoint-modulating regimens) demonstrates that certain tumor and immune programs remain pharmacologically modifiable; thus, epigenetic therapies may act most effectively as selective strategies for destabilizing the HRS state, increasing immune visibility, and enhancing proximity to apoptosis—in this sense, the HRS phenotype may not be fully reversible, yet its dependence on continuously reinforced and stably maintained epigenetic circuitry renders it therapeutically vulnerable.

## Figures and Tables

**Table 1:** Representative molecular mechanisms stabilizing the HRS cell state in classical Hodgkin lymphoma and their relative reversibility.

Mechanism	Representative Target/State	Functional Consequence	Relative Reversibility	Key References
DNA methylation *	*POU2AF1*, *CD79B*, *TCL1A*, and other B-cell promoters	Stable repression of B-cell-associated transcription	**Intermediate → low**; lower plasticity with dense methylation	[30,31]
Histone deacetylation	B-cell-associated promoters including *CD19*, *MS4A1*, and *CD79B*	Reduced chromatin accessibility and B-cell gene expression	**Higher relative plasticity**, but reacetylation/demethylation does not restore full B-cell identity	[4,5]
Polycomb repression	PRC2/EZH2–H3K27me3 and PRC1/RING1–H2AK119ub1; abnormal BMI1/RYBP expression	Reinforcement of durable transcriptional repression	**Low relative plasticity**, particularly when combined with DNA methylation	[4,21,31,39,42,43]
Transcription factor disruption	E2A/*TCF3* antagonism by ABF-1 and Id2; impaired PAX5-dependent circuitry	Loss of B-lineage transcriptional programs	**Incomplete reversibility demonstrated**; restoring individual factors is insufficient	[17,18,19]
Histone demethylase dysregulation	JMJD2C/KDM4C, KDM6B, KDM4B/KDM4D	Altered H3K9/H3K27 methylation, differentiation programs, and treatment-associated phenotypes	**Uncertain**	[6,55,57]
*CREBBP*/*EP300* and *ARID1A* lesions	Histone acetyltransferase and chromatin remodeling functions	Genetically entrenched disruption of transcriptional/chromatin regulation	**Genetically fixed**; downstream effects may remain pharmacologically modifiable	[62,64,69]
9p24.1 amplification	*JAK2*, *CD274*/PD-L1, and *PDCD1LG2*/PD-L2	Increased JAK/STAT activity and checkpoint ligand expression	**Genetically fixed lesion; modifiable downstream output**	[65,66,67,68]
Antigen presentation disruption	*B2M*, *HLA-B*, and *CIITA*	Reduced MHC class I/II expression and immune visibility	**Fixed when structural; potentially modifiable when suppression is regulatory**	[64,75,76,77]

* Relative reversibility represents an interpretive hierarchy derived from the framework discussed in Section 3.4 rather than a validated quantitative classification. “Higher relative plasticity” denotes chromatin states that are experimentally or pharmacologically modifiable; “intermediate” and “low” denote progressively more stable, multilayered repression; and “genetically fixed” denotes structural genomic alterations that cannot themselves be reversed by epigenetic therapy, although downstream transcriptional or signaling consequences may remain therapeutically modifiable. Reversibility is locus- and context-dependent.

**Table 2:** Phase II studies of epigenetic and epi-immunotherapeutic strategies in relapsed/refractory classical Hodgkin lymphoma.

Regimen	Class	Population	*n*	Response	Survival/Durability
Panobinostat [107] *	Pan-HDACi	R/R cHL after ASCT	129	ORR 27%; CR 4%	PFS 6.1 mo; DOR 6.9 mo; 1-year OS 78%
Mocetinostat [108]	Class I/IV HDACi	R/R cHL	51	ORR 27.5%; CR 3.9%	Median PFS/OS not reported
Entinostat [112]	Class I HDACi	R/R cHL after ASCT or ASCT-ineligible	49	ORR 12%; CR 0%	PFS 5.5 mo; OS 25.1 mo; DOR 28.5 mo in responders
Decitabine + camrelizumab [118,120]	DNMTi + anti-PD-1	R/R cHL, PD-1-naïve	42	ORR 95%; CR 79%	PFS 35.0 mo; 2-year PFS 67%; DOR NR
Decitabine + camrelizumab [118]	DNMTi + anti-PD-1	R/R cHL after prior PD-1 therapy	25	ORR 52%; CR 28%	Responses > 6 mo in 10 patients
Chidamide + decitabine + camrelizumab [121]	Class I/IIb HDACi + DNMTi + anti-PD-1	R/R cHL after decitabine + camrelizumab	52	ORR 94%; CR 50%	PFS 29.4 mo

* Cross-trial comparisons are descriptive because patient populations and follow-up differed. The 42-patient decitabine–camrelizumab row represents the combination arm of the PD-1-naïve randomized cohort; ref. [120] provides extended follow-up of the cohort initially reported in [118]. Abbreviations: ASCT, autologous stem cell transplantation; cHL, classical Hodgkin lymphoma; CR, complete response; DNMTi, DNA methyltransferase inhibitor; DOR, duration of response; HDACi, histone deacetylase inhibitor; NR, not reached; ORR, objective response rate; OS, overall survival; PD-1, programmed cell death protein 1; PFS, progression-free survival; R/R, relapsed/refractory.

## Data Availability

No new data were created or analyzed in this study. Data sharing is not applicable to this article.

## Abbreviations

The following abbreviations are used in this manuscript:

5hmC	5-Hydroxymethylcytosine
5mC	5-Methylcytosine
ABF-1	Activated B-cell factor 1
AICE	AP-1-IRF composite element
AP-1	Activator protein 1
BCR	B-cell receptor
bHLH	Basic helix–loop–helix
CAF	Cancer-associated fibroblast
CE	Composite element
cHL	Classical Hodgkin lymphoma
ChIP	Chromatin immunoprecipitation
ChIP-chip	Chromatin immunoprecipitation coupled to promoter microarrays
*CIITA*	Class II major histocompatibility complex transactivator
CR	Complete remission
ctDNA	Circulating tumor DNA
DNMT	DNA methyltransferase
EBV	Epstein–Barr virus
EV	Extracellular vesicle
FDC	Follicular dendritic cell
GC	Germinal center
HDAC	Histone deacetylase
HLH	Helix–loop–helix
HLA	Human leukocyte antigen
HRS	Hodgkin and Reed–Sternberg
Ig	Immunoglobulin
IgH	Immunoglobulin heavy chain
IgL	Immunoglobulin light chain
IgVH	Immunoglobulin variable heavy-chain
lncRNA	Long non-coding RNA
LMP1	Latent membrane protein 1
LMP2A	Latent membrane protein 2A
MAPK/ERK	Mitogen-activated protein kinase/extracellular signal-regulated kinase
MGZL	Mediastinal gray zone lymphoma
MHC	Major histocompatibility complex
miRNA	MicroRNA
NF-κB	Nuclear factor kappa B
NGS	Next-generation sequencing
NK	Natural killer
NS-cHL	Nodular sclerosis classical Hodgkin lymphoma
OS	Overall survival
PD-1	Programmed cell death protein 1
PD-L1	Programmed death ligand 1
PD-L2	Programmed death ligand 2
PFS	Progression-free survival
PMBCL	Primary mediastinal large B-cell lymphoma
PRC1	Polycomb repressive complex 1
PRC2	Polycomb repressive complex 2
RS	Reed–Sternberg
siRNA	Small interfering RNA
TAM	Tumor-associated macrophage
TARC	Thymus and activation-regulated chemokine
TCR	T-cell receptor
TET	Ten-eleven translocation
Tfh	Follicular T helper
TMA	Tissue microarray
TME	Tumor microenvironment
Treg	Regulatory T cell
TSA	Trichostatin A
